# Monkeypox in South Asia: a systematic review

**DOI:** 10.1080/22221751.2025.2572677

**Published:** 2025-10-08

**Authors:** Kinley Wangdi, Ripon Adhikary, Ziqi Liu, Sotiris Vardoulakis, Rosemary A. McFarlane, Manas Kotepui, Wenbiao Hu, Apiporn T. Suwannatrai, Tsheten Tsheten

**Affiliations:** a HEAL Global Research Centre, Health Research Institute, Faculty of Health, University of Canberra, Bruce, Australia; b National Centre for Epidemiology and Population Health, Australian National University, Acton, Australia; c Medical Technology, Faculty of Science, Nakhon Phanom University, Nakhon Phanom, Thailand; d School of Public Health and Social Work, Queensland University of Technology, Kelvin Grove, Australia; e Department of Parasitology, Faculty of Medicine, Khon Kaen University, Khon Kaen, Thailand; f Ageing and Aged Care Analysis Unit, Australian Institute of Health and Welfare, Bruce, Canberra, Australia

**Keywords:** Mpox, South Asia, epidemiology, disease severity, transmission, public health response

## Abstract

Monkeypox (Mpox) has emerged as a significant public health concern globally and in South Asia. Therefore, this systematic review aimed to provide a synthesis of the epidemiological pattern, clinical features, disease severity, and mitigation strategies in the region. A systematic review was conducted across four databases including PubMed, Scopus, Web of Science, and Ovid from inception to August 2024. Out of 2,430 studies, only 19 met the inclusion criteria with 111 confirmed Mpox cases. Forty percent (44) of patients were males and 45.9% (51) did not specify their sex. Young to middle-aged adults were most affected, with rare cases in a neonate. Around two-thirds (73.0%, 81) of Mpox patients were from India, followed by Pakistan (16.2%, 18) and Nigeria (resident in India) (9.9%, 11). Sixty-eight percent (76) of Mpox patients reported recent travel, particularly to UAE (27%, 21) and Saudi Arabia (26.3%, 20). Common signs and symptoms among cases were skin lesion (92.8%, 103), fever (82.0%, 91), lymphadenopathy (65.8%, 73), muscle ache (27.0%, 30), and genital and perianal lesions (17.1%, 19). Co-infection with herpes simplex virus and varicella zoster virus led to difficulty in diagnosis. Nearly all the studies used polymerase chain reaction for diagnosis. Public health responses varied across countries, including enhanced surveillance, contact tracing, and awareness campaigns, but vaccine availability remained limited. Mpox in South Asia was largely linked to travel-related transmission and primarily affected younger adults. Strengthening surveillance systems, a syndemic approach, diagnostic capacity, and targeted interventions are crucial to controlling its spread.

## Introduction

Monkeypox is caused by the Monkeypox virus (MPXV; hereinafter referred to as Mpox), which belongs to the *Orthopoxvirus* genus of the Poxviridae family [1]. It was first identified at a polio research facility in Copenhagen in 1958 in cynomolgus monkeys (*Macaca fascicularis*), shipped from Singapore [2]. However, the first human cases were reported in Central and West Africa in the 1970s [3]. Zoonotic infection is associated with multiple wild mammals in Africa [2]. Over the years, sporadic outbreaks have been reported in non-endemic regions primarily due to human-to-human transmission. Additionally, international trade and travel have contributed to the spread of the infection, where as importation of infected animals has been rarely implicated [4]. Mpox in humans has an incubation period of 5–21 days and symptoms include fever, a vesicular – pustular eruption and lymphadenopathy [5–7]. It has an estimated mortality rate of 10.6% in Central Africa and <3.6% in West Africa [8, 9]. The primary mode of transmission from person to person is contact with infectious lesions or respiratory droplets. Transmission through seminal fluid has been reported [10, 11].

While historically confined to endemic regions in Africa, cases have now been reported in multiple non-endemic regions including Europe, America and Asia. Since the World Health Organization (WHO) declared Mpox a public health emergency on 23 July 2022 [12], 126 countries have reported cases [5]. Between January 2022 and August 2024, WHO reported over 10,000 laboratory confirmed cases and 220 deaths among confirmed cases [13]. In the WHO South-East Asia Region (SEAR), there were 959 cases and 11 deaths reported as of September 2024 [14]. India was the first country in SEAR to report Mpox, on 12 July 2022 [15]. Since then, many countries in the region have reported Mpox cases including deaths [16, 17]. The index cases of Mpox were acquired overseas with subsequent cases infected locally [14]. Despite ongoing efforts, the disease continues to spread, particularly in densely populated areas with limited healthcare resources.

South Asia has experienced a steady increase in Mpox cases, many linked to individuals returning from endemic areas. However, challenges such as delayed diagnosis, insufficient public awareness, and inadequate healthcare infrastructure have hindered timely response efforts [17]. Given the region’s high population density and interconnected travel networks, there is a pressing need for robust surveillance mechanisms to prevent further outbreaks and mitigate the disease’s impact. Understanding the epidemiology and clinical profile of Mpox in South Asia is crucial for improving case identification, optimizing treatment protocols, minimizing transmission risk, and enhancing patient outcomes [18].

As the region prepares for Mpox, there is a paucity of information regarding Mpox cases in the South Asia. This systematic review aimed to provide a comprehensive analysis of Mpox cases in South Asia by examining epidemiological trends, clinical characteristics, disease severity, and public health responses. By synthesizing available evidence, this study seeks to identify knowledge gaps, inform policy development, and supports efforts to enhance regional preparedness and outbreak response.

## Methods

### Search strategy

A comprehensive search strategy was developed to identify peer-reviewed studies reporting Mpox cases in South Asia including Afghanistan, Bangladesh, Bhutan, India, Maldives, Nepal, Pakistan, and Sri Lanka. The search was conducted across four major databases: PubMed, Scopus, Web of Science, and Ovid from inception to August 2024. The strategy utilized two domains: Domain 1 (D1), focused on Mpox-specific terms such as “monkeypox,” “mpox,” “monkey pox,” and “Orthopoxvirus,” and Domain 2 (D2), targeted geographic terms relevant to South Asia, including “Afghanistan,” “Bangladesh,” “Bhutan,” “India,” “Maldives,” “Nepal,” “Pakistan,” “Sri Lanka,” and “Asia.” Boolean operators were used to combine the domains (D1 AND D2), ensuring search results focused on studies linking Mpox with the South Asian region.

Search fields were restricted to titles and abstracts in PubMed, TITLE-ABS-KEY in Scopus, TS (Topic) in Web of Science, and keyword mapping (.mp) in Ovid to ensure a comprehensive and precise search. Full search strings were tailored to each database to optimize retrieval accuracy. The detailed search terms used for each database are shown in Supplementary Table 1.

Results were exported into EndNote version 20.0 (Clarivate Analytics, Philadelphia, PA, USA) for deduplication, and unique entries were imported into Rayyan for systematic review management. Studies were initially screened based on titles and abstracts. Studies selected by title and abstract underwent full text screening for the final selection. Reference lists of included studies were also hand-searched to identify additional relevant articles.

### Eligibility criteria

To ensure relevance, inclusion and exclusion criteria were applied rigorously. The PICO (Population, Intervention, Context, and Outcome) framework used to identify eligible studies for this review is included in Supplementary Table 2. We included studies involving humans of all age/sex categories. Studies were excluded if they only involved animals, in-vitro, reviews or those in language other than English.

### Study selection

A two-stage selection process was employed. In the first stage, two independent reviewers (RKA and ZL) screened the titles and abstracts of all retrieved articles to assess eligibility based on the eligibility criteria. Articles considered potentially relevant proceeded to full-text screening in the second stage. Disagreements during any stage were resolved through discussion or by consulting a third reviewer (KW). Finally, reference lists of selected studies were manually reviewed to ensure comprehensive inclusion of relevant articles.

### Data extraction

Data from included studies were extracted using a standardized extraction form. The form was piloted on three studies to ensure consistency between reviewers, and included fields such as study characteristics Author and reference, country, cases, age (years), comorbidity, methods of diagnosis, mode of transmission, intervention, recommendations. Data extraction was independently performed by two reviewers (RKA and ZL) and cross-checked for accuracy. Discrepancies were resolved through team discussion or involvement of a third reviewer (KW).

### Risk of bias and data synthesis

The methodological quality of the included studies was assessed using the Joanna Briggs Institute (JBI) critical appraisal checklists appropriate for analytical cross-sectional, case–control, and cohort studies [19]. The full risk of bias in individual studies was not assessed because of heterogeneity in study design including case reports, viewpoints, and mini review. Extracted data were synthesized descriptively and presented in tabular and narrative formats. Quantitative findings were tabulated to summarize study characteristics, clinical presentations, and public health responses. Narrative synthesis was employed to analyse qualitative findings, such as risk factors, clinical outcomes, and research gaps. We used ArcGIS Pro version 3.3 (ESRI, Redlands, CA) to create a choropleth map by countries to indicate the number of studies retrieved and included from each country. The graphs were created using Rstudio Desktop Version: 2025.05.1 (R Foundation for Statistical Computing, Vienna, Austria).

## Results

### Study selection

**Figure 1.**
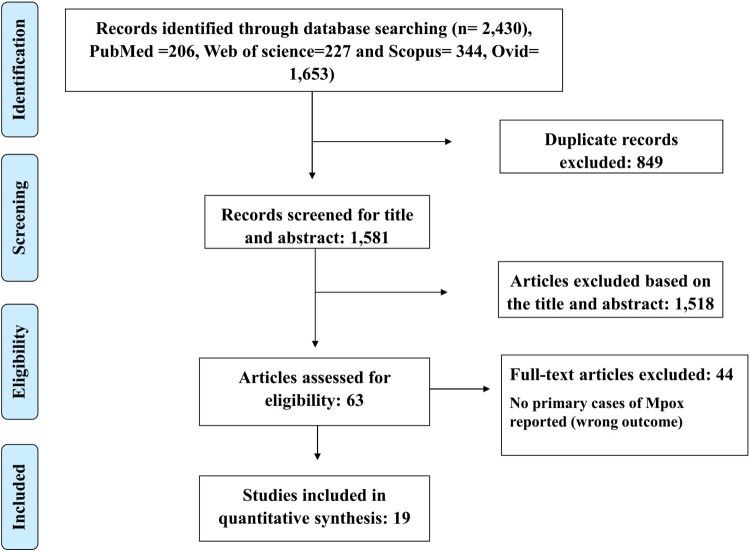
PRISMA flow chart for study selection.

From the four electronic databases including PubMed, Scopus, Web of Science, and Ovid, a total of 2,430 articles were retrieved. After removing 849 duplicates, 1,581 records were screened based on title and abstract. Then 1,518 records were removed based on title and abstract leaving 63 articles. Forty-four articles did not meet the eligibility criteria and were excluded after reviewing full text. Nineteen studies were included in the final analysis (Figure 1).

### Study characteristics

**Figure 2.**
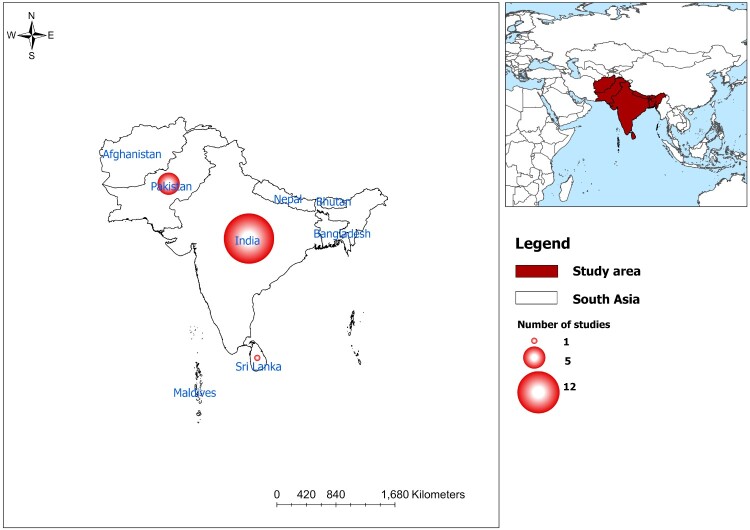
Study locations with bubbles size corresponding to the number of studies reviewed from inception to August 2024. Only 18 studies are presented here with one study based on multi-country.

**Figure 3.**
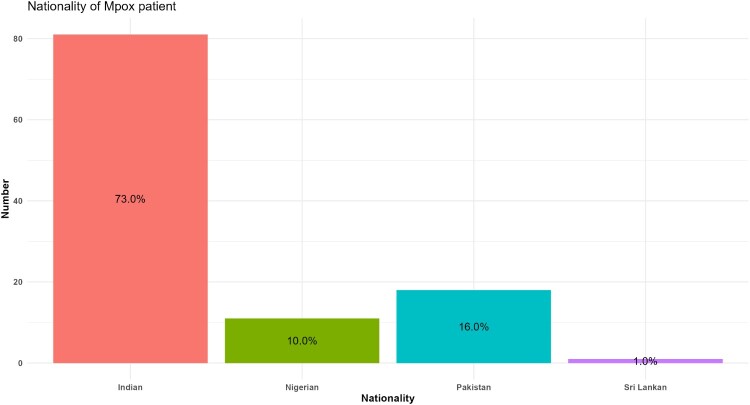
Distribution of Mpox patients by nationality.

Of the 19 studies [20–38], 12 (68.4%) were from India [20, 22, 25–28, 31, 32, 34–37] followed by Pakistan [21, 23, 29, 30, 33], Sri Lanka [24], and multi-country [38] (with data from India) (Figure 2). In total, there were 111 confirmed cases with two deaths [29, 32]. Nearly half of the confirmed cases were males (44, 39.6%), and 14.4% (16) were females, while 45.9% (51) did not specify their gender. Nearly two-thirds (81, 73.0%) of Mpox patients were from India, followed by Pakistan (18, 16.2%) and Nigeria (11, 9.9%) and one from Sri Lanka (1, 0.9%) (Figure 3). Mpox cases in South Asia predominantly affected young to middle-aged adults, typically between 22 and 35 years. The youngest patient was a neonate while the oldest patient was 60 years old (Table 1). The first confirmed case was reported in India in 2022 involving a 35-year-old male traveller from the United Arab Emirates (UAE). The first Mpox-related death was also reported in India in a 22-year-old male with encephalitis [37] followed by a 40-year-old Pakistani man due to complications from human immunodeficiency virus (HIV) [29]. While most cases were among adults, exceptions included neonatal case, such as an 8-day-old infant from Sri Lanka who contracted Mpox from close family members [24].

**Table 1.** Demographic characteristics of the included studies from South Asia.

Author and reference	Country	Cases	Age (years)	Comorbidity	Methods of diagnosis	Mode of transmission	Intervention	Recommendations
George et al. 2023 [20]	India	1	1	HSV, transaminitis	PCR	Human-to-human	Isolation of the patient	Early detection of patients
Mallhi et al. 2023 [21]	Pakistan	5	Adults	NR	PCR	Human-to-human, travel-related	Screening at entry points	Improve healthcare preparedness
Mohapatra et al. 2022 [22]	India	10	NS	NR	RT-PCR, serology	Human-to-human	Screening and awareness campaigns	Accurate differentiation between Mpox and HFMD
Mughal et al. 2023 [23]	Pakistan	5	Adults	NR	PCR	Human-to-human	Public health alert issued	Need for a syndemic response
Pattiyakumbura et al. 2023 [24]	Sri Lanka	1	Neonate	NR	PCR, X-ray	Mother-to-neonate transmission	Neonatal care; oxygen therapy	Awareness for neonatal cases
Relhan et al. 2022 [25]	India	5	Mean 31	Hepatitis B (one case)	PCR	Human-to-human	Isolation and testing centres established	Enhanced community surveillance
Sahay et al. 2023 [27]	India	8	Adults	NR	PCR	Surface-to-human potential	Rigorous disinfection protocols in isolation wards	Surface decontamination
Sahoo et al. 2022 [28]	India	12	Median 28	HIV (one case)	PCR	Human-to-human, sexual contract	Conservative treatment; condom usage post-recovery	Prevention of STI-related transmission
Sah et al. 2022 [26]	India	4	Adults	NR	PCR, serology	Human-to-human	Contact tracing; isolation	Strengthened surveillance in SEAR
Satapathy et al. 2024 [29]	Pakistan	7	NS	HIV	PCR	Human-to-human, travel-related	Limited interventions; screening	Public health education
Shabbir et al. 2023 [30]	Pakistan	2	Adults	NR	PCR	Human-to-human	Quarantine measures, awareness campaigns	Improved screening at entry points
Sharma et al. 2023 [31]	India	1	22	NR	PCR, genome sequencing	Human-to-human	Genome sequencing	Early warning systems
Singh et al. 2022 [32]	India	10	21–31	One fatal case with encephalitis	PCR	Human-to-human	Expanded isolation units	Inclusion of vaccination protocols
Umair et al. 2023 [33]	Pakistan	4	16,18,32,34	VZV	PCR	Human-to-human	Case isolation; genomic sequencing	Awareness for high-risk groups
Vasu et al. 2023 [34]	India	10	Mean 33	One fatal case with encephalitis	PCR	Human-to-human	Testing and monitoring protocols	Address stigma in health-seeking
William et al. 2023 [35]	India	10	22–35	NR	PCR	Human-to-human	Testing in isolated hospitals	Management of asymptomatic cases
Yadav et al. 2022 [36]	India	2	31 &35	NR	PCR, genome sequencing	Human-to-human, close contact	Case isolation and contact tracing	Strengthened surveillance of travellers
Yadav et al. 2023 [37]	India	1	22	NR	PCR, genome sequencing	Human-to-human	Supportive ICU care	Recognition of atypical presentations
Yang et al. 2023 [38]	Multi-country	12	NS	NR	Secondary data	Human-to-human	Surveillance of global cases	Research on stigma effects

NS – Not specified; NR – not reported; HSV – herpes simplex virus; PCR – polymerase chain reaction; VZV – varicella zoster virus; HFMD – Hand foot and mouth disease; ICU – intensive care unit; STI – sexually transmitted infection; SEAR – WHO South-East Asia Region.

### Travel history and other modes of transmission

**Figure 4.**
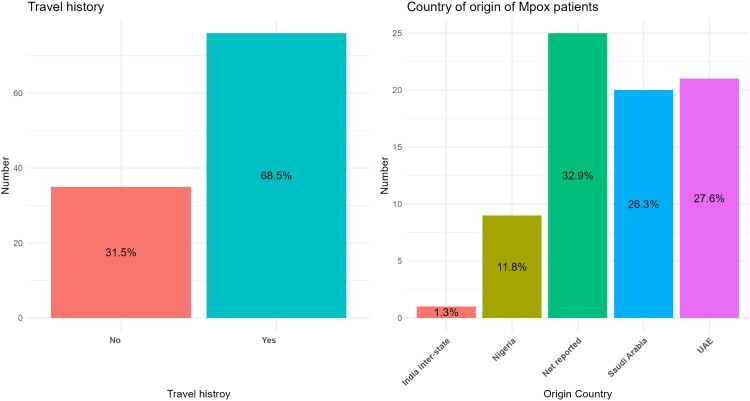
Travel history and country of travel amongst Mpox patients in the South Asia region.

Sixty-eight (76, 68.5%) patients reported a history of travel in the last three weeks prior to the diagnosis (Figure 4 and Supplementary Table 3). UAE was the most common destination of travel with 27% (21) of patients followed by Saudi Arabia (20, 26.3%) and Nigeria (9, 11.8%). However, 32.9% (25) did not report the country of travel (Figure 4).

The common mode of transmission was person-to-person. Several studies, including those from India and Pakistan, emphasized the role of close personal and sexual contact in spreading the virus. Notably, cases in India involving Nigerian nationals residing in Delhi were potentially linked to intimate contact or shared living spaces [25]. Neonatal transmission was reported in Sri Lanka, where a newborn contracted the virus from her mother and possibly her father, both of whom had symptoms after returning from Saudi Arabia [24] (Table 1).

Fomite transmission through contaminated surfaces was identified as a potential risk factor in healthcare and household settings. A study from India demonstrated the persistence of Mpox virus on surfaces in hospital isolation wards, emphasizing the need for rigorous disinfection and hygiene practices to mitigate secondary infections [27]. Healthcare workers also faced exposure risks due to environmental contamination and improper use of personal protective equipment (PPE) in isolation wards, as demonstrated in hospital-based studies in New Delhi [27]. Additionally, certain population groups, such as men who have sex with men (MSM), have shown higher prevalence rates of Mpox [21, 39] (Table 1).

### Clinical features and disease severity

**Figure 5.**
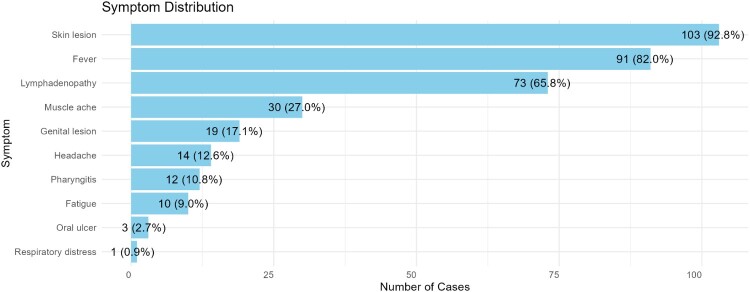
Symptoms of Mpox in the South Asia region. *Skin rash included vesicle, pustule, and macule.

Common signs and symptoms among confirmed cases were vesiculo-pustular rashes (103, 92.8%,, fever (91, 82.0%), lymphadenopathy (73, 65.8%), and muscle aches (30, 27.0%). The rash typically progressed through stages, starting as macules, progressing to papules, vesicles, pustules, and finally scabs, with some studies reporting centrifugal distribution, initially affecting the face before spreading to other body parts, including the palms and soles. Atypical lesions that localized predominantly in the genital and perianal regions were also presented in 17.1% (19) of patients. Oral ulcers and follicular tonsillitis were observed in 10.8% (12) of patients (Figure 5).

Rare but severe complications, such as meningoencephalitis, have been reported in South Asia. For example, a fatal case in Kerala, India, involved a 22-year-old male with the clinical manifestation of fever, seizures, and altered sensorium, eventually progressing to acute brainstem dysfunction [32]. This case highlights the potential for Mpox to cause severe neurological involvement. Mpox in neonate (Sri Lanka) was associated with complications, including bronchopneumonia, emphasizing the need for specialized care in vulnerable populations [24].

Comorbidities such as HIV infection, have been identified as contributing factors to disease severity. For instance, the first fatal case of Mpox in Pakistan involved a 40-year-old man with HIV who succumbed to encephalitis [29]. Co-infections with other viruses, such as herpes simplex virus (HSV) and varicella zoster virus (VZV), have also been reported, leading to diagnostic challenges and influencing clinical outcomes [31, 33]. Immunocompromised individuals were particularly vulnerable to severe Mpox [33].

### Risk of bias

Of 19 studies, only four studies qualified for risk of bias assessment – two each for case reports [20, 24] and case series [25, 28]. For case reports, studies clearly described demographic characteristics, patients’ history, current clinical conditions of patients, diagnostic methods, and treatment. The post-interventional conditions, adverse events, and takeaway lessons were clearly described in the papers (Supplementary Table 4). Similarly, case studies outlined clear inclusion criteria, conditions were reliably measured, consecutive and complete inclusion of participants. The demographics, clinical information of the participants were clearly outlined. The outcomes or follow up results of cases were clearly reported. Statistical analysis done where they were appropriate (Supplementary Table 5).

### Diagnosis and source of infection

Accurate and timely diagnosis of Mpox is essential for effective case management and control of transmission. Nearly all the studies employed polymerase chain reaction (PCR) and genomic analysis [20–37] except for one study that used secondary data, and diagnostic methods were not reported [38]. Two studies reported the sources of samples for diagnosis: these were blood, urine, and lesion swabs of body fluids [20, 25] (Table 1).

### Public health responses

**Figure 6.**
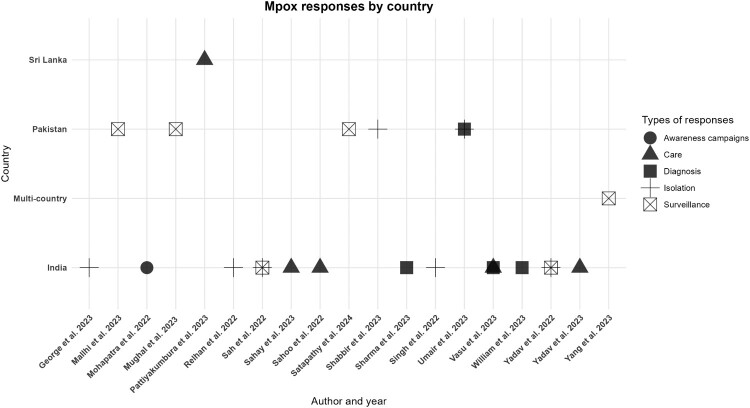
Country-specific interventions for Mpox in the South Asia region.

The emergence of Mpox in South Asia has prompted varied governmental responses across countries including awareness campaigns, guidelines for managing (care) of Mpox patients, diagnosis, isolation protocols, and surveillance. However, responses varied across countries. India implemented all five recommended measures, while Pakistan effectively carried out surveillance, isolation, and diagnosis but fell short in conducting widespread awareness campaigns. While Sri Lanka reported only implementing guidelines for managing Mpox patients (Figure 6 and Table 1).

While no specific Mpox vaccine has been deployed widely in South Asia, governments have considered vaccination for high-risk groups. India's public health guidelines discuss the potential role of smallpox vaccines, given their cross-protective efficacy against Mpox [35]. However, logistical challenges and limited vaccine availability have hindered large-scale implementation. Therefore, the capacity to manufacture vaccines locally would greatly improve South Asia vaccine access [40].

In Pakistan, the National Institute of Health (NIH) spearheaded surveillance and monitoring efforts, particularly at points of entry such as airports, where travellers were screened for symptoms. Educational campaigns were conducted to address stigma associated with Mpox, particularly among vulnerable populations such as the MSM community [21]. These campaigns aimed to encourage early reporting and reduce barriers to seeking healthcare. Sri Lanka adopted similar strategies, integrating Mpox monitoring into existing surveillance frameworks for other infectious diseases [24].

## Discussion

This systematic review offers a comprehensive synthesis of current knowledge on Mpox in South Asia, highlighting key epidemiological patterns, clinical presentations, disease severity, and mitigation measures. In the region, Mpox cases were frequently reported among travelers returning from the Middle East and other international destinations. Human-to-human transmission and stigma associated with Mpox resulted in delayed diagnosis and poor compliance with isolation. All cases reported similar clinical manifestations to those reported in other areas. Mpox response in the region were multifaceted but enhanced surveillance and education were two important measures that contained outbreaks. The findings highlight critical gaps in research and response strategies, emphasizing the need for region-specific responses.

Mpox cases in South Asia have predominantly been linked to travel from endemic regions, particularly the Middle East, with a smaller proportion involving local transmission [21, 27]. This trend highlights the critical role of cross-border mobility in the spread of Mpox and underscores the importance of effective screening at points of entry [41]. While India has reported the highest number of cases in the region, other countries like Pakistan and Sri Lanka have also documented cases, suggesting the need for robust surveillance systems. In 2025, global Mpox cases continue to be reported from Africa including Gambia, Sierra Leone, Guinea, Liberia, Ghana, and Togo [42]. Cases have decreased in the Americas to 902 cases and one death in nine countries with Brazil (425 cases and 1 death) and the United States (245) reporting the most cases from the region [42]. In Europe, 117 cases were reported between April and May 2025, mostly by Germany [33], the Netherlands [21], Spain [14], and France [13, 43].

In South Asia, Mpox cases have predominantly been reported among young to middle-aged adults, reflecting global trends in disease epidemiology [44]. However, rare instances involving a neonate has also been documented, such as the case from Sri Lanka, highlighting the potential vulnerability of this age group despite their low representation in overall case counts [24]. This neonatal case underscores the importance of strengthening maternal and child health surveillance within Mpox surveillance systems to ensure early detection and appropriate clinical management.

Human-to-human transmission remains the primary mode of spread in the region, while contaminated fomites and potential sexual transmission were other sources of infection, similar to findings from other parts of the world [44, 45]. Several studies identified certain population, including MSM, people who engage in higher-risk sexual practices, and immunocompromised patients, such as those with HIV, were at a higher risk of Mpox infection [21, 29]. The stigma associated with these groups may delay diagnosis and treatment, reduce compliance with isolation and other preventive measures, and result in reluctance to disclose contacts among infected individuals, further exacerbating transmission risks [46]. Misconceptions about Mpox, and the perceived link to specific sexual behaviours has deterred individuals from seeking medical care, potentially leading to spreading of disease in communities [38]. This is especially relevant in cases involving MSM communities, where stigma may have amplified delays in seeking healthcare access [25].

The clinical presentation of Mpox in South Asia aligns with global patterns, with commonly reported symptoms including vesiculo-pustular rashes, fever, lymphadenopathy, and myalgia [20, 34]. However, several studies documented atypical manifestations such as genital lesions, localized rashes, and neurological complications, including encephalitis [33, 37]. These findings highlight the need for healthcare providers to recognize diverse presentations to avoid misdiagnosis, particularly in settings with overlapping diseases such as VZV or HFMD [47].

Similar to other studies, this study reported that the pre-existing comorbidities, such as HIV, diabetes, and hypertension were associated with disease severity [48]. Additionally, co-infections with other viral pathogens such as HSV and VZV were documented, further complicating clinical outcomes [33, 49]. These findings reinforce the importance of early identification and targeted management strategies for populations with existing comorbidities, including immunocompromised individuals and neonates.

Government responses to Mpox in South Asia were multifaceted with various strategies used to contain outbreaks. Enhanced surveillance and screening measures, particularly at airports and points of entry, have played a crucial role in identifying and isolating cases early. In India, specific guidelines were issued for healthcare workers to facilitate symptom recognition and management [30, 32], while in Pakistan, educational campaigns targeted stigma reduction, particularly among high-risk groups such as MSM [25, 38]. Despite these efforts, the absence of a widely deployed Mpox vaccine in the region remains a critical gap [21, 50]. To date, there is no proven effective antiviral treatment for Mpox. Some antivirals have received emergency use authorization in some countries and are being evaluated in clinical trials. It is a priority to continue evaluation and to focus on optimizing supportive care for patients [51]. While smallpox vaccines have been considered for high-risk groups, challenges such as limited vaccine availability and logistical barriers have hindered large-scale implementation. In addition, all studies failed to outline the public acceptance of vaccine. Therefore, strengthening vaccination strategies by overcoming the logistical barriers, bolstering diagnostic capacity, prioritization of vaccine strategies, and public acceptance should be explored. Furthermore, addressing social stigma through public awareness campaigns are essential components of an effective Mpox mitigation plan in South Asia.

Polymerase chain reaction and genomic analysis were the commonly used diagnostic methods using swabs from lesions and body fluids [20–37]. In the absence of skin lesions, testing can be done using swabs of the throat or anus. Other methods including immunohistochemistry of skin lesions and electron microscopy, which can provide important clinical evidence [52]. However, PCR or genetic sequence remains a gold standard for the diagnosis of Mpox. So, strengthening these diagnostic capabilities should be undertaken for future management of Mpox in the region.

This systematic review is limited by the available studies, which varied in their scope, methodology, and reporting quality. Many studies lacked detailed demographic, clinical, and temporal data. These may have influenced the findings of this review. Potentially this is due to studies predominantly reporting case reports or case series with limited ability of the disease to cause widespread local infection. Future research should prioritize standardized data collection and reporting to facilitate regional and global comparisons.

The analysis of Mpox in South Asia highlights several significant research gaps that must be addressed to enhance disease management and control. To strengthen Mpox preparedness and response, we recommend enhancing early detection through improved lesion recognition, community-based surveillance, screening at entry points, surveillance of travellers, and the establishment of robust early warning systems. Healthcare preparedness should be reinforced by ensuring accurate differentiation between Mpox and similar conditions such as HFMD, recognizing atypical and asymptomatic presentations, and incorporating vaccination protocols where feasible. The role of animal reservoirs in zoonotic transmission also remains poorly understood [50]. Public health responses should prioritize a syndemic approach, surface decontamination, and prevention of STI-related transmission. Awareness campaigns must address neonatal and high-risk groups, promote public health education, and reduce stigma that hinders health-seeking behaviours. Finally, collaborative efforts addressing these gaps can support evidence-based policy, control and preparedness for Mpox in South Asia.

## Conclusion

Mpox is becoming a serious public health problem in the region with some countries reporting locally acquired cases alongside the ongoing risk of importation due to international travel and trade. Addressing the research gaps and enhancing diagnostic, surveillance, and response capabilities will be critical to mitigating the disease's impact in the region. A coordinated approach involving cross-border collaboration, community engagement and education, and investment in healthcare infrastructure is required to improve Mpox management, prevention and preparedness. Future public health strategies must incorporate strengthened surveillance, improved diagnostic capacity, culturally sensitive response and targeted interventions to reduce transmission in high-risk groups and ensure effective outbreak control in South Asia.

## Author contributions

KW, TT, and RA were involved in conceptualization, formal analysis, supervision, validation, visualization, writing – original draft, reviewing, and editing. RA and ZL were involved in data curation, investigation, and writing – review and editing. SV, RM, MK, WH, and ATS were involved in supervision and critical revision of manuscript. All authors approved the final draft.

## Supplementary Material

Supplementary file.docx
