# Supplementary material for: Monkeypox in South Asia: a systematic review
Source: Emerg Microbes Infect. 2025 Oct 8;14(1):2572677. doi: 10.1080/22221751.2025.2572677 (PMC12541925; doi:10.1080/22221751.2025.2572677)
Supplement: Supplementary file.docx [file TEMI_A_2572677_SM3885.docx]

**Supplementary Tables**

**Supplementary Table 1. The search strategies for different database.**

| Databases | Search Strings |
| --- | --- |
| PubMed | ("monkeypox*"[Title/Abstract] OR "mpox*"[Title/Abstract] OR "monkey pox"[Title/Abstract] OR "orthopoxvirus*"[Title/Abstract]) AND ("asia*"[Title/Abstract] OR "Afghanistan"[Title/Abstract] OR "Bangladesh"[Title/Abstract] OR "Bhutan"[Title/Abstract] OR "Himalayas"[Title/Abstract] OR "India"[Title/Abstract] OR "Maldives"[Title/Abstract] OR "Nepal"[Title/Abstract] OR "Pakistan"[Title/Abstract] OR "Sri Lanka"[Title/Abstract]) |
| Scopus | (TITLE-ABS-KEY (monkeypox* OR mpox* OR "monkey pox" OR orthopoxvirus*)) AND (TITLE-ABS-KEY (asia* OR afghanistan OR bangladesh OR bhutan OR himalayas OR india OR maldives OR nepal OR pakistan OR "Sri Lanka")) |
| Web of Science | TS= (monkeypox* OR mpox* OR "monkey pox" OR orthopoxvirus*) [AND] TS= (asia* OR afghanistan OR bangladesh OR bhutan OR himalayas OR india OR maldives OR nepal OR pakistan OR "Sri Lanka") |
| Ovid | (monkeypox* or mpox* or "monkey pox" or orthopoxvirus*).mp. [mp=ti, ab, tx, ct, sh, bt, ot, hw, cw, nm, fx, kf, ox, px, rx, an, ui, ds, on, sy, ux, mx, tc, id, tm, mf] [AND] (asia* or afghanistan or bangladesh or bhutan or himalayas or india or maldives or nepal or pakistan or "Sri Lanka").mp. [mp=ti, ab, tx, ct, sh, bt, ot, hw, cw, nm, fx, kf, ox, px, rx, an, ui, ds, on, sy, ux, mx, tc, id, tm, mf] |

**Supplementary Table 2. Inclusion and exclusion applied to studies using Population, Intervention, Context and Outcome (PICO) strategy*.***

| Parameter | Inclusion criteria | Exclusion criteria |
| --- | --- | --- |
| Population | Human | Non-human species |
| Intervention | Infected with Mpox virus | Other than Mpox virus |
| Context | South Asian (SA) countries | Other than SA |
| Outcome | Mpox cases/reporting | Other than Mpox cases |

**Supplementary Table 3. Spatio-temporal distribution and origin of Mpox in South Asia region**

| **Study & reference** | **Country** | **Spatial pattern** | **First Case Date** | **Cross-border (Yes/No)** | **Country of origin** |
| --- | --- | --- | --- | --- | --- |
| George et al. 2023 (20) | India | Kerala: First case localized in Trivandrum | May-22 | Yes | UAE |
| Mallhi 2023 (21) | Pakistan | Major urban centers | Apr-23 | Yes | Saudi Arabia |
| Mohapatra et al. 2022 (22) | India | Kerala, Delhi, susp in Telangana | Jul-22 | Yes | Middle East |
| Mughal et al. 2023 (23) | Pakistan | Cases isolated at entry points | May-23 | Yes | Saudi Arabia |
| Pattiyakumbura 2023 (24) | Sri Lanka | Western Province | Nov-22 | Yes | Saudi Arabia |
| Relhan et al. 2022 (25) | India | Localized cases in Delhi | Jun-22 | No | NA |
| Sahay et al. 2023 (27) | India | Isolation ward surface contamination | Aug-22 | No | NA |
| Sahoo et al. 2022 (28) | India | North India clusters | Jul-22 | Yes | Nigeria |
| Sah et al. 2022 (26) | India | Kerala, Delhi | Jul-22 | Yes | UAE |
| Satapathy et al. 2024 (29) | Pakistan | Scattered cases across districts | Apr-23 | Yes | Saudi Arabia |
| Shabbir et al. 2023 (30) | Pakistan | Isolated to Islamabad, Karachi | Apr-23 | Yes | Saudi Arabia |
| Sharma et al. 2023 (31) | India | Single case in Delhi | Mar-23 | No | NA |
| Singh et al. 2022 (32) | India | Kerala and Delhi | Jul-22 | Yes | UAE |
| Umair et al. 2023 (33) | Pakistan | Urban spread in Pakistan | Apr-23 | Yes | Saudi Arabia |
| Vasu et al. 2023 (34) | India | Localized to Kerala | Jul-22 | Yes | UAE |
| William et al. 2023 (35) | India | Kerala and Delhi | Jul-22 | Yes | UAE |
| Yadav et al. 2022 (36) | India | Kerala localized outbreak | Jul-22 | Yes | UAE |
| Yadav et al. 2023 (37) | India | Kerala encephalitis case | Jul-22 | Yes | UAE |
| Yang et al. 2023 (38) | Multi-country | Broad spread across Asia-Pacific | Jan-22 | Yes | UAE, USA, Europe |

**Supplementary Table 4. Quality assurance of case reports.**

| **Reference** | **Country** | 1. Were patient’s demographic characteristics clearly described? | 2. Was the patient’s history clearly described and presented as a timeline? | 3. Was the current clinical condition of the patient on presentation clearly described? | 4. Were diagnostic tests or assessment methods and the results clearly described? | 5. Was the intervention(s) or treatment procedure(s) clearly described? | 6. Was the post-intervention clinical condition clearly described? | 7. Were adverse events (harms) or unanticipated events identified and described? | 8. Does the case report provide takeaway lessons? |
| --- | --- | --- | --- | --- | --- | --- | --- | --- | --- |
| George et al. 2023 (20) | India | Yes | Yes | Yes | Yes | Yes | Yes | Yes | Yes |
| Pattiyakumbura et al. 2023 (24) | Sri Lanka | Yes | Yes | Yes | Yes | Yes | Yes | Yes | Yes |

**Supplementary Table 5. Quality assurance of case studies.**

| **Reference** | **Country** | 1. Were there clear criteria for inclusion in the case series? | 2. Was the condition measured in a standard, reliable way for all participants included in the case series? | 3. Were valid methods used for identification of the condition for all participants included in the case series? | 4. Did the case series have consecutive inclusion of participants? | 5. Did the case series have complete inclusion of participants? | 6. Was there clear reporting of the demographics of the participants in the study? | 7. Was there clear reporting of clinical information of the participants? | 8. Were the outcomes or follow up results of cases clearly reported? | 9. Was there clear reporting of the presenting site(s)/clinic(s) demographic information? | 10. Was statistical analysis appropriate? |
| --- | --- | --- | --- | --- | --- | --- | --- | --- | --- | --- | --- |
| Relhan et al. 2022 (25) | India | Yes | Yes | Yes | Yes | Yes | Yes | Yes | Yes | Yes | Yes |
| Sahoo et al. 2022 (28) | India | Yes | Yes | Yes | Yes | Yes | Yes | Yes | Yes | Yes | Yes |
